# Surgical Management of Pediatric Obstructive Sleep Apnea: Efficacy, Outcomes, and Alternatives—A Systematic Review

**DOI:** 10.3390/life14121652

**Published:** 2024-12-12

**Authors:** Gianna Dipalma, Angelo Michele Inchingolo, Irene Palumbo, Mariafrancesca Guglielmo, Lilla Riccaldo, Roberta Morolla, Francesco Inchingolo, Andrea Palermo, Ioannis Alexandros Charitos, Alessio Danilo Inchingolo

**Affiliations:** 1 Department of Interdisciplinary Medicine, University of Bari “Aldo Moro”, 70121 Bari, Italy; angeloinchingolo@gmail.com (A.M.I.); m.guglielmo2@studenti.uniba.it (M.G.); l.riccaldo@studenti.uniba.it (L.R.); roberta.morolla@uniba.it (R.M.); ad.inchingolo@libero.it (A.D.I.); 2 Department of Experimental Medicine, University of Salento, 73100 Lecce, Italy; andrea.palermo@unisalento.it; 3 Pneumology and Respiratory Rehabilitation Unit, Istituti Clinici Scientifici Maugeri IRCCS, 70124 Bari, Italy; ioannis.charitos@icsmaugeri.it

**Keywords:** obstructive sleep apnea, OSAS, surgical treatment, tonsillectomy, pediatric OSA

## Abstract

Aim: Obstructive sleep apnea (OSA) is the most prevalent sleep-related breathing disorder. OSA affects approximately 2 million Italians, although only 3% receive a diagnosis and correct treatment. This review aims to provide an overview to guide clinical decision making, ensuring that patients receive the most appropriate treatment for their specific condition. Material and Methods: This systematic review was conducted according to the Preferred Reporting Items for Systematic Reviews and Meta-Analyses (PRISMA) guidelines and registered at PROSPERO under the ID CRD42024593760. A search on PubMed, Scopus, and Web of Science was performed to find papers that matched the topic, using the following Boolean keywords: (“obstructive sleep apnea” OR “OSA” OR “sleep apnea, obstructive”) AND (“surgery” OR “surgical” OR “surgical techniques” OR “surgical treatment” OR “operative” OR “surgical procedures”) AND (“treatment” OR “therapy” OR “management”). Result: The electronic database search found 20337 publications. After the screening and eligibility phase, 15 papers were chosen for the qualitative analysis. Conclusions: Adenotonsillectomy (AT) significantly improves secondary outcomes like behavioral issues and quality of life, compared to watchful waiting with supportive care (WWSC). Alternative approaches such as tonsillotomy and adenopharyngoplasty (APP) offer promising results, with less postoperative discomfort and lower complication rates. However, further large-scale studies are needed to refine surgical techniques, assess long-term outcomes, and optimize individualized treatment strategies for OSA.

## 1. Introduction

**Figure 1:**
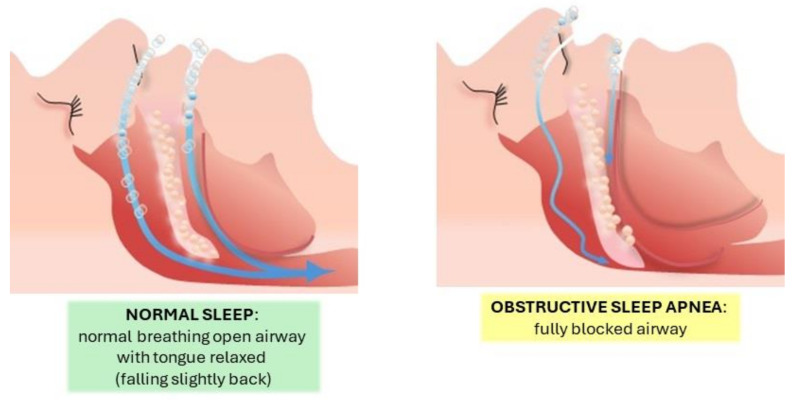
Graphic representation of the difference in airflow during normal breathing in sleep versus obstructive sleep apnea.

Sleep apnea is a condition characterized by pauses in breathing during sleep that last more than ten seconds and can vary in duration (Figure 1) [1,2,3,4,5,6,7,8].

There are three types of sleep apnea:

Obstructive Sleep Apnea (OSA): OSA is characterized by the blockage of the upper airway despite ongoing respiratory efforts. This leads to a stop in oro-nasal airflow while thoracic and abdominal respiratory movements persist [9,10,11,12,13,14,15,16,17,18,19].
Central Sleep Apnea (CSA): This type occurs when there is a cessation of airflow without any respiratory effort due to a temporary lack of neural signals to the respiratory muscles. It is marked by a simultaneous halt in oro-nasal airflow and thoraco-abdominal respiratory movements [9,20,21,22,23,24,25,26,27,28].
Mixed or Complex Apnea: This type starts with a cessation of airflow without respiratory effort, similar to central apnea, but progresses with increasing respiratory effort [29,30,31,32,33,34,35,36,37].

OSA is the most prevalent sleep-related breathing disorder. First described in 1965, OSA affects approximately 2 million Italians, although only 3% receive a diagnosis [38,39,40,41,42]. The syndrome is identified by two key features: numerous prolonged apneas exceeding 15 events per hour throughout sleep and reduced blood oxygen saturation [43,44,45,46,47]. The diagnosis of OSA is typically made when there is a combination of significant blood oxygen desaturation and an apnea–hypopnea index (AHI) greater than 10.

Hypopnea is defined as a partial reduction in airflow during sleep, resulting in a decrease in respiratory effort of at least 30% for a duration of at least 10 s, accompanied by a reduction in oxygen saturation of 3% or more, or arousal from sleep. It represents a less severe form of airway obstruction compared to apnea, where there is a complete cessation of airflow.

OSA severity can be classified based on the AHI and the lowest oxygen saturation level (Nadir SpO2) reached during sleep [48,49,50,51]. Common symptoms include snoring and daytime sleepiness, which can impact both social and family life due to decreased cognitive performance. Statistical studies indicate a higher incidence of car accidents caused by drowsiness among patients with OSA. This is attributed to the non-restorative sleep they experience, characterized by frequent micro-awakenings, leading to persistent fatigue throughout the day [52,53,54,55,56,57]. In children, who can also suffer from this syndrome often due to adeno-tonsillar obstruction, symptoms may include learning difficulties, restlessness, aggression, and poor academic performance [58,59,60,61,62,63]. The condition can be exacerbated, particularly in obese individuals, by severe complications affecting vital organs, especially the cardiovascular and central nervous systems [64,65,66].

### 1.1. Diagnosis

Diagnosing OSA involves a comprehensive clinical evaluation and specific instrumental assessments [67,68,69,70,71,72].

Otolaryngological evaluation is used to determine the location and extent of upper airway obstruction.

Polysomnography is a multi-channel recording of physiological signals during sleep to evaluate sleep architecture and diagnose sleep disorders [73]. It typically includes the continuous monitoring of electroencephalography, electrooculography, electromyography, electrocardiography, respiratory effort, airflow, oxygen saturation (SpO2), and leg movements. The purpose of PSG is to observe and quantify various physiological parameters during sleep [74,75,76,77].

Specialist consultations in cardiology, pulmonology, neurology, endocrinology, pediatrics, sexology, dentistry, and psychology may be necessary to evaluate and manage complications related to OSA [78,79,80,81,82,83,84,85,86,87,88].

### 1.2. Treatment

Treatment involves a multidisciplinary approach, encompassing both medical and surgical interventions.

#### 1.2.1. Medical Therapy

The first step includes lifestyle changes to reduce factors that contribute to snoring, such as alcohol, smoking, sedatives, and overeating. Weight loss is crucial to reduce fat deposits in the throat structures. Limited pharmacological options are available, mainly aimed at improving nasal ventilation [74,89,90,91].

#### 1.2.2. Positional Therapy

Positional therapy is a non-invasive treatment option for patients. This therapy aims to prevent or reduce apnea events by encouraging the patient to sleep in non-supine positions, such as on their side or stomach, often using devices like positional pillows or vests. It is particularly beneficial for mild-to-moderate cases but is less effective for severe OSA or multi-level airway obstructions. Positional therapy is commonly used in combination with other treatments [92].

#### 1.2.3. Mechanical Therapy

Among mechanical treatments, oxygen therapy improves oxygen saturation but does not reduce the number or duration of apneas. The most effective non-surgical treatment is the use of a continuous positive airway pressure (CPAP) device, which maintains continuous airflow through a nasal mask during the night, preventing apnea episodes. CPAP is recommended even in conjunction with surgical procedures in severe cases [93,94,95,96].

#### 1.2.4. Oral Appliance Therapy

Particularly useful for habitual snorers with significant positional (supine) components, this involves using portable orthodontic devices at night to reposition the mandible and tongue, thereby enlarging the airway space. These appliances can be used alone or in combination with surgery and CPAP, with their use being determined through sleep endoscopy [94,97,98,99,100].

#### 1.2.5. Surgical Therapy

Various surgical procedures aim to improve nasal ventilation and correct anatomical abnormalities associated with snoring. The choice of surgery depends on the clinical profile and site of obstruction [101,102,103,104,105].

Adenoidectomy, with or without tonsillectomy (TC), is often sufficient in children [106,107,108,109,110,111].

TC, with or without additional palate surgery, is recommended for adults with large, obstructive tonsils [112,113,114,115,116].

Adenotonsillectomy (AT) involves the removal of both the tonsils and adenoids, which are often enlarged and contribute to airway obstruction. In the context of OSA, early AT is typically performed in young children, usually before the age of 3–4, especially those with severe or refractory symptoms, such as recurrent infections or significant nocturnal hypoxia or apnea, which may affect their growth and development.

Routine AT is generally performed at an older age, typically during childhood or adolescence, for children with moderate OSA or recurrent episodes of upper airway infections. This approach is planned and performed when the child’s symptoms become chronic or recurrent, with the goal of improving quality of life and reducing the frequency of OSA episodes [117].

Septoplasty is the realignment of a deviated nasal septum to restore nasal patency, often combined with turbinectomy or turbinate reduction surgery [118,119,120].

Another approach is the use of minimally invasive surgery for the soft palate, uvula, and base of the tongue.

Techniques like radiofrequency ablation, snoreplasty, and pillar implants aim to stiffen or retract the soft palate, reducing snoring and vibration. These procedures are reserved for simple snoring or mild OSAS [121].

Anterior or lateral pharyngoplasty remodels the soft palate and uvula without tissue removal, reducing the risks associated with older uvulopalatoplasty techniques.

Laryngeal surgery includes procedures like epiglottidectomy or hyoid suspension, which are performed under general anesthesia for cases involving epiglottic obstruction or lateral pharyngeal collapse [122].

The array of surgical options ensures that treatment can be tailored to the individual patient’s needs and the specific nature of their airway obstruction [123,124,125,126].

The purpose of this systematic review is to analyze the best surgical techniques for treating OSA in pediatric patients. Additionally, it aims to determine whether surgical intervention is truly necessary or if less invasive alternatives could provide similar benefits. Given the delicate nature of pediatric patients, it is crucial to identify therapeutic approaches that maximize clinical efficacy while minimizing risks and complications. The goal is to provide a comprehensive, evidence-based overview to guide clinical decision making, ensuring that patients receive the most appropriate treatment for their specific condition.

## 2. Materials and Methods

### 2.1. Protocol and Registration

The protocol was registered at PROSPERO with the ID CRD42024593760, and the systematic review was carried out in accordance with the Preferred Reporting Items for Systematic Reviews and Meta-Analyses (PRISMA) guidelines.

### 2.2. Search Processing

Papers related to the surgical management of OSA in pediatric patients were found by searching PubMed, Scopus, and Web of Science between 1 January 2009 and 1 June 2024. “Obstructive sleep apnea” OR “OSA” OR “sleep apnea, obstructive” AND “surgery” OR “surgical” OR “surgical techniques” OR “surgical treatment” OR “operative” OR “surgical procedures”) AND (“treatment” OR “therapy” OR “management”) were the Boolean keywords utilized in the search strategy (Table 1).

### 2.3. Inclusion Criteria

The following were the inclusion criteria: (1) open access studies; (2) studies that examined the use of surgery to treat OSA in children; (3) randomized clinical trials, retrospective studies, case–control studies, and prospective studies; (4) English language; and (5) full-text studies.

Papers that did not match the above criteria were excluded.

The review was conducted using the following PICOS criteria:

Participants: children, both male and female;
Interventions: use of surgery in the treatment of OSA;
Comparisons: alternative approach to watchful waiting with supportive care;
Outcomes: AT remains a cornerstone in the treatment of pediatric OSA caused by adenotonsillar hypertrophy (ATH);
Study: randomized clinical trials, retrospective studies, case–control studies, and prospective studies.

### 2.4. Exclusion Criteria

The following were the exclusion criteria: (1) animal research; (2) unrelated topics; (3) reviews, case reports, case series, correspondence, or remarks; and (4) language other than English.

### 2.5. Data Processing

Based on the selection criteria, three reviewers (M.G., I.P., and R.M.) independently searched the databases to gather the studies and assessed their quality. Version 6.0.15 (Corporation for Digital Scholarship, Vienna, VA, USA) was used to download the chosen articles. A senior reviewer (F.I.) was consulted in order to resolve any disagreements among the three writers.

### 2.6. Quality Assessment

The quality of the included papers was assessed by three reviewers—M.G., I.P., and R.M.—using the ROBINS tool [127].

A degree of bias was assessed for each of the seven points that were evaluated. If there was dispute, a third reviewer (F.I.) was consulted until a consensus was reached. The following appeared among the domains assessed by ROBINS:

- confounding bias;
- bias resulting from exposure measurement;
- bias in a study’s participant selection;
- bias resulting from post-exposure intervention;
- bias resulting from missing data;
- bias resulting from outcome measurement,
- bias in the presentation of the results.

## 3. Results

### 3.1. Study Selection and Methodological Features

The electronic database search provided 20,337 publications in total (Scopus N = 7644, PubMed N = 7097, and Web of Science N = 5596); the manual search yielded no articles.

**Figure 2:**
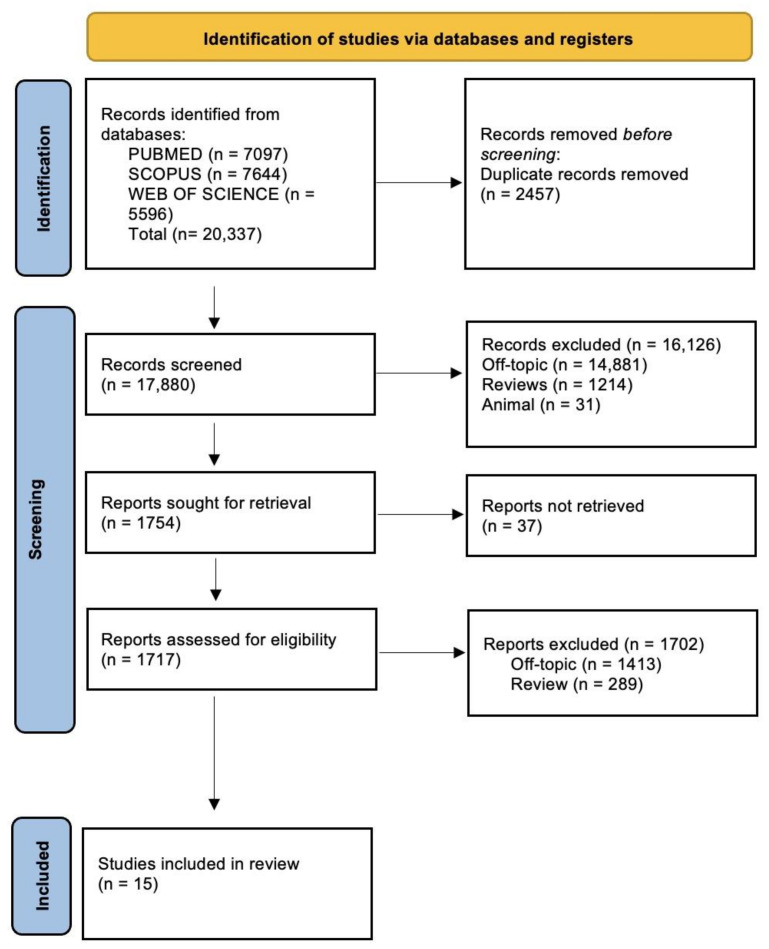
Literature search following the Preferred Reporting Items for Systematic Reviews and Meta-Analyses (PRISMA) flow diagram and database search indicators.

After duplicates (N = 2457) were eliminated, 17,880 studies’ titles and abstracts were evaluated for screening. Out of these, 16,126 papers did not meet the inclusion criteria (14,881 off-topic, 1214 reviews, and 31 animal studies), leaving 1754 records to be selected for the review. Out of these, 1702 reports were removed for not meeting the inclusion criteria (1413 off-topic, 289 reviews), and 37 records could not be collected. After being determined to be eligible, fifteen records were selected for qualitative analysis. The selection process is shown in Figure 2, and the summary of the records that were chosen is shown in Table 2.

AT remains effective for moderate-to-severe pediatric OSA, improving neurocognitive, behavioral, and quality-of-life outcomes, particularly in obese children. However, in mild OSA cases, spontaneous AHI normalization has raised questions about the necessity of surgery. The impact of AT on cognitive function varies, with some studies showing benefits for sleep quality but limited effects on higher cognitive functions.

Studies on AT techniques have shown mixed results. Some report no significant impact on growth, while others have found higher cure rates with modifications like tonsillar pillar closure, although the differences are not statistically significant. Alternative techniques, such as coblation, have been shown to reduce operative time and postoperative pain without affecting long-term outcomes. In terms of comorbidities, improvements in cardiac autonomic function have been noted, though their clinical significance remains uncertain. Alternatives to AT, like ATT, offer faster recovery but higher recurrence rates. Some studies report a 15% recurrence rate in patients with ATT. The combination of APP and AT has been shown to result in less postoperative bleeding and faster healing, without affecting speech. Comparisons of AT and APP have shown similar reductions in AHI, with AT being preferred for more severe cases. Additionally, combining RME with AT has demonstrated benefits in cases with maxillary constriction, though further research is needed to optimize treatment sequencing.

### 3.2. Study Characteristics

The results from these 15 studies on pediatric obstructive sleep apnea syndrome (OSA) reveal a range of significant improvements across various outcomes. Behavioral changes, sleepiness, symptoms, quality of life, blood pressure, and a reduction in the progression of the apnea–hypopnea index (AHI) were noted. In particular, the adenotonsillectomy (AT) group showed notable improvements, with 79% of patients achieving normalization in behavior, quality of life, and polysomnography indices. Early AT significantly reduced monotonous heart rate patterns during quiet sleep and temporarily increased the body mass index (BMI) and weight in children, although no final difference in BMI was observed between early and routine surgery groups.

Coblation AT was associated with shorter operative times, less intraoperative blood loss, and reduced postoperative pain compared to traditional dissection methods. While a higher cure rate was observed in the pillar closure group, this was not statistically significant. Spontaneous AHI normalization occurred in 40% of the WWSC group, though persistent inspiratory effort remained. Improvements in attention, executive functioning, and polysomnography indices were also reported. The plasma group experienced lower postoperative pain and improvements in BMI and cognitive function, with no significant differences in immune response markers.

Long-term follow-up data (5 years) suggested that ATT could be effective in treating pediatric OSA, while no significant differences between the groups were observed in some outcomes. The combination of APP and TC resulted in a shortened healing period and reduced blood loss without increasing postoperative complications. Six months post surgery, the OM-6 scores improved significantly in both groups, though no differences were observed in audiological outcomes. Overall, both treatments led to significant improvements in clinical symptoms, polysomnographic findings, and quality of life, including reductions in sleep disturbances and improvements in VAS quality-of-life scores.

### 3.3. Quality Assessment and Risk of Bias of Included Articles

**Figure 3:**
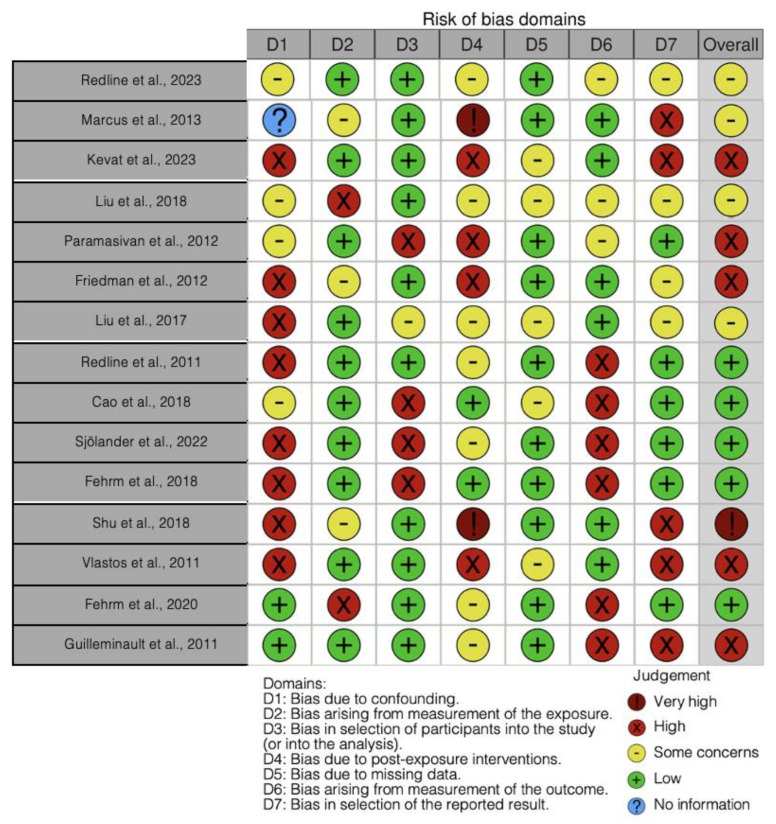
Bias assessment [61,62,63,64,65,66,67,68,69,70,71,72,73,74,75].

In the analysis of the 15 studies, the overall risk of bias varied significantly: 5 studies showed low risk, 4 presented some concerns, 5 had high risk, and 1 study demonstrated a very high risk of bias. When analyzing the individual domains, bias due to confounding was predominantly high, with eight studies classified as high risk, four having some concerns, two presenting low risk, and one study providing no information. The bias arising from the measurement of exposure generally showed a low risk, with 10 studies assessed as low risk, 1 study having some concerns, and 4 studies presenting a high risk. Regarding the bias in the selection of participants, most studies (10) presented a low risk, while 1 showed some concerns, and 4 exhibited a high risk of bias. For the bias due to post-exposure interventions, two studies had a low risk, seven studies showed some concerns, four had a high risk, and two were rated as very high risk. The bias due to missing data was mostly low, with 10 studies classified as low risk and 5 showing some concerns. Bias arising from the measurement of the outcomes was more evenly distributed, with six studies rated as low risk, three with some concerns, and six presenting a high risk. Lastly, the bias in the selection of the reported results showed six studies with low risk, four with some concerns, and five with high risk (Figure 3).

## 4. Discussion

### 4.1. Efficacy of AT

AT is considered the primary treatment for moderate-to-severe pediatric OSA, but uncertainties remain regarding its long-term efficacy, especially in mild cases. The CHAT trial by Redline et al. (2011) demonstrated significant improvements in neurocognitive, behavioral, and quality-of-life parameters in children undergoing early AT, particularly among obese individuals and those from ethnic minorities [135]. However, other studies, such as Liu et al. (2017), reported that the spontaneous normalization of AHI may occur without surgical intervention, raising the question of whether AT is necessary for all children with mild OSA [134].

Subsequent studies highlighted the variable efficacy of AT. Kevat et al. observed a temporary increase in BMI during the first postoperative year, followed by normalization in the second year, with no significant impact on height growth between children undergoing early or delayed intervention [130]. Furthermore, Friedman et al. (2012) explored the effect of tonsillar pillar closure during AT, reporting higher cure rates (89.5% vs. 72%) compared to the standard technique. However, this difference was not statistically significant, emphasizing the need for larger-scale studies [133]. Additionally, Paramasivan et al. compared the coblation technique to traditional dissection, identifying advantages of coblation in terms of shorter operative times, reduced blood loss, and lower postoperative pain, while maintaining comparable long-term safety and outcomes [132].

### 4.2. Outcomes of AT

AT provides benefits in reducing OSA severity and improving quality of life, but its effects on other comorbidities and physiological parameters are variable. Liu et al. (2017) reported that, while AT reduces thoraco-abdominal asynchrony across all sleep phases, it does not always lead to clinically significant improvements compared to WWSC in the long term [134]. The CHAT trial demonstrated significant improvements in neurocognitive and behavioral parameters in children with OSA following AT, although the efficacy was not uniform across all higher cognitive functions [135]. Regarding neurocognitive outcomes, studies by Redline et al. and Marcus et al. showed that, while AT significantly benefits quality of life and behavior, it does not consistently improve higher cognitive functions compared to conservative management [128,129]. Cao et al. (2018) highlighted that the use of low-temperature plasma tonsillectomy reduces postoperative pain and improves cognitive and social parameters without compromising the immune response [136]. The impact of AT on OME, however, remains controversial. Previous studies reported that adenoidectomy can improve middle ear function and reduce the need for ventilation tubes, but findings regarding improvements in OME and hearing are inconsistent. This underscores the importance of further research in this area [131,140]. In terms of cardiac function, Liu et al. demonstrated improvements in heart rate variability modulation in children with OSA following AT. However, it remains unclear whether these benefits have long-term clinical significance, as similar improvements were observed in the WWSC group [134].

### 4.3. Alternatives to AT

Among alternatives to AT, ATT stands out for its faster recovery times and lower postoperative pain, although it carries a higher risk of OSA recurrence. A prospective study conducted at the Karolinska University Hospital reported that 15% of patients undergoing ATT required reintervention due to recurrences, highlighting the need for long-term follow-up [137]. APP combined with AT represents another viable option. A study at the Children’s Hospital of Chongqing Medical University found that patients undergoing APP with AT had lower rates of postoperative hemorrhage (0.91% vs. 4.36%) and faster healing times compared to standalone AT, without adverse effects on velopharyngeal function or speech [139]. RME has also proven effective in cases of OSA associated with reduced maxillary width, especially when combined with AT. A study demonstrated that both techniques contribute to symptom resolution, suggesting a sequential approach for complex cases [142]. Finally, a study at the Karolinska University Hospital compared AT and APP, finding similar reductions in AHI (88% for AT and 91% for APP) six months postoperatively. Both techniques are effective, but AT remains the preferred treatment for more severe cases [138,141]. Emerging surgical techniques for pediatric OSA, such as low-temperature plasma tonsillectomy and coblation, show promise in reducing complications and improving recovery, but their long-term effects and risks, like residual OSA or tonsillar regrowth, require further study. Techniques like APP combined with TC have demonstrated benefits, including lower hemorrhage rates and faster healing, though larger trials are needed to confirm their role in clinical practice.

For children with mild OSA, spontaneous improvement in respiratory symptoms and AHI is common, especially in younger patients. While early surgery can improve quality of life and behavior, the CHAT trial suggests that not all children with mild OSA require immediate intervention. Identifying which patients benefit most from surgery versus conservative management remains crucial for optimizing outcomes.

## 5. Limitations

The limitations of this systematic review involve several aspects. First, the small sample sizes may not have been sufficient to detect significant differences in cure rates or functional recovery, indicating the need for larger studies to validate the findings. Additionally, the variability in outcomes, such as AHI normalization and behavioral improvements, highlights the complexity of predicting patient responses to AT versus WWSC, suggesting that the results may not be universally applicable. Although some studies reported serious adverse events related to surgery, the low percentage of affected children limits the ability to generalize these risks to broader populations. Another area of uncertainty concerns the optimal sequence of combined treatments, such as using AT with RME, pointing to the need for further research to determine the best approach for individual patients. Finally, mixed results regarding the impact of adjuvant therapies, such as adenoidectomy on conditions like OME effusion, suggest that additional studies are needed to better understand their role and effectiveness in conjunction with AT.

## 6. Conclusions

AT is an effective solution for moderate-to-severe pediatric OSA, with clear benefits in terms of symptom reduction, quality of life, and behavioral parameters. However, its efficacy in mild cases and younger children remains an area of investigation. Surgical alternatives, such as ATT, APP, and RME, offer advantages for specific patient subgroups but require careful treatment personalization. Coblation and low-temperature plasma tonsillectomy appear to be promising options for reducing complications and improving postoperative recovery. Optimizing therapeutic strategies for pediatric OSA necessitates further large-scale studies to balance the benefits of surgery against associated risks and the potential for spontaneous improvement, ensuring personalized, evidence-based treatments.

## Figures and Tables

**Table 1:** Full search strings for each database.

PubMed	(“obstructive sleep apnea” OR “OSA” OR “sleep apnea, obstructive”) AND (“surgery” OR “surgical” OR “surgical techniques” OR “surgical treatment” OR “operative” OR “surgical procedures”) AND (“treatment” OR “therapy” OR “management”)
Scopus	(“obstructive sleep apnea” OR “OSA” OR “sleep apnea, obstructive”) AND (“surgery” OR “surgical” OR “surgical techniques” OR “surgical treatment” OR “operative” OR “surgical procedures”) AND (“treatment” OR “therapy” OR “management”)
Web of Science	(“obstructive sleep apnea” OR “OSA” OR “sleep apnea, obstructive”) AND (“surgery” OR “surgical” OR “surgical techniques” OR “surgical treatment” OR “operative” OR “surgical procedures”) AND (“treatment” OR “therapy” OR “management”)

**Table 2:** Descriptive summary of item selection.

Author (Year)	Study Design	Number of Patients	Average Age and Gender	Surgical Technique	Outcomes
Redline et al., 2023 [128]	RCT	459	Mean age, 6.1 years; 230 female (50%)	Adenotonsillectomy (AT)	Improvements in behavior, sleepiness, symptoms, quality of life, blood pressure, and reduced AHI progression.
Marcus et al., 2013 [129]	RCT	464 children	5 to 9 years; gender not specified	Early AT	Significant improvements in behavior, quality of life and polysomnography, with 79% normalization in the AT group.
Kevat et al., 2023 [130]	RCT	190 children	Aged 3–5 years; gender not specified	AT (early or routine)	AT temporarily increased body mass index (BMI) and weight in children, with no final BMI difference between early and routine surgery groups.
Liu et al., 2018 [131]	RCT	354 children	Not specified	Early AT vs. WWSC	Early AT significantly reduced monotonous heart rate patterns during quiet sleep.
Paramasivan et al., 2012 [132]	Prospective comparative study	100 children	5 to 12 years; gender not specified	Coblation AT versus dissection method	Coblation AT was associated with a shorter operative time, less intraoperative blood loss, and reduced postoperative pain compared to the dissection method.
Friedman et al., 2012 [133]	Prospective randomized, single-blind, controlled	60 patients (25 completed standard tonsillectomy(TC) and adenoidectomy; 19 completed pillar closure	Mean age: 9.0 ± 4.3 years (standard T&A); 8.9 ± 3.8 years (pillar closure); 16 male and 14 female per group	Cold-steel TC and adenoidectomy with or without tonsillar pillar closure using 3.0 chromic suture	Higher cure rate observed in pillar closure group, but not statistically significant.
Liu et al., 2017 [134]	Randomized controlled trial (RCT)	353 children	Mean age: 6.6 years; 49% male	Complete bilateral TC and adenoidectomy using standard surgical techniques	Spontaneous AHI normalization observed in 40% of the WWSC group, but with persistent inspiratory effort.
Redline et al., 2011 [135]	Randomized controlled trial (RCT)	464 children	Mean age: 7.0 years; mixed gender	Early AT	Significant improvements in attention, executive functioning, and polysomnography indices.
Cao et al., 2018 [136]	Two-center randomized controlled trial	72 children (36 study group, 36 control group)	Study group: 5.3 ± 1.4 years; 20 males and 16 females. Control group: 5.2 ± 1.3 years; 21 males and 15 females	Low-temperature plasma TC (study group) vs. traditional tonsil-pecking method (control group)	The plasma group had lower postoperative pain and improved BMI and cognitive function, with no differences in immune response markers.
Sjölander et al., 2022 [137]	Prospective single-center randomized trial	79	Age: 2–6 years; gender: 53 M 26 F	Comparison between AT and adenotonsillotomy (ATT)	After 5 years of follow-up, ATT could be effective in treating pediatric OSA.
Fehrm et al., 2018 [138]	RCT	83	Mean age: 36.6 months; gender: 49 males (59%) and 34 females (41%)	Comparison between AT and Adenopharyngoplasty (APP)	No significant difference between the groups.
Shu et al., 2018 [139]	RCT	603	Group APP+TC: 328 (138 F, 190 M), mean age: 4.9 ± 2.1 years. TC group: 275 (108 F, 167 M), mean age 5.13 ± 2.4 years	Comparison between APP and TC vs. TC alone	APP combined with TC shortened the healing period and decreased blood loss but did not result in an increase in postoperative problems.
Vlastos et al., 2011 [140]	RCT	52	Mean age 4.5 years; 56% M, 44% F	Group one received adenoidectomy plus tympanostomy tube insertion, while group two received adenoidectomy plus myringotomy	Six months post surgery, OM-6 scores improved significantly in both groups, with no differences in audiological outcomes.
Fehrm et al., 2020 [141]	Prospective RCT	60	2 to 4 years ATE group—52% male; WWSC group—61% male	ATE involving blunt extracapsular dissection for tonsils and adenoid removal with a ring knife or coblation	Significant improvements in quality of life, sleep disturbance, and VAS quality of life
Guilleminault et al., 2011 [142]	Preliminary randomized study	31	Average age 6.5; 14 M and 17 F	ATE and rapid maxillary expansion (RME) or bimaxillary expansion	Significant improvement in clinical symptoms and polysomnography findings after both treatments.

## Abbreviations

AHI	Apnea–hypopnea index
APP	Adenopharyngoplasty
AT	Adenotonsillectomy
ATH	Adenotonsillar hypertrophy
ATT	Adenotonsillotomy
BMI	Body mass index
CHAT	Childhood adenotonsillectomy trial
CPAP	Continuous positive airway pressure
CSA	Central sleep apnea
OME	Otitis media with effusion
OSA	Obstructive sleep apnea
RME	Rapid maxillary expansion
TAA	Thoraco-abdominal asynchrony
TC	Tonsillectomy
WWSC	Watchful waiting with supportive care
